# Comparative efficacy of muscle energy technique and Bowen technique on hamstrings muscle tightness in chronic low back pain patients

**DOI:** 10.12669/pjms.40.9.8517

**Published:** 2024-10

**Authors:** Kishwar Batool, Mariam Mehmood, Muneeb Jafar, Maham Gull

**Affiliations:** 1 Kishwar Batool, The University of Faisalabad, Faisalabad, Pakistan; 2 Mariam Mehmood, The University of Faisalabad, Faisalabad, Pakistan; 3 Muneeb Jafar, The University of Faisalabad, Faisalabad, Pakistan; 4 Maham Gull, The University of Faisalabad, Faisalabad, Pakistan

**Keywords:** Flexibility, Muscle tightness, Lower back pain, Randomized clinical trial

## Abstract

**Objective:**

To compare the effects of the Muscle Energy Technique (MET) and the Bowen Technique on hamstring muscle tightness in chronic low back pain (CLBP) patients.

**Method:**

A randomized clinical trial (RCT) designed study in which 62 participants were recruited through the purposive sampling technique were divided into two groups by the lottery method. Subjects who had pain for more than six months in the back and hamstring tightness were included. The duration of the study was four months from February to May 2023 conducted at Madinah Teaching Hospital Faisalabad. Subjects in Group-A were given the Bowen technique, whereas subjects in Group-B were given the MET. Numeric pain rating scale (NPRS), Oswestry disability index (ODI), and active knee extension (AKE) tests were used for measurements of outcomes.

**Results:**

Intra-group comparison by using Friedman test revealed that both Group-A & B subject’s pain were reduced (P<0.000), their tightness in back thigh muscles were significantly reduced (P<0.000) and functional activities of subjects were also improved (P<0.000). Mann Whitney test was used to determine the between group comparison. P-value ≤ 0.05 was considered as significant. Inter Group-Analysis revealed that there was no statistically significant difference between effects of Bowen technique & MET on pain, hamstring tightness and disability as both techniques improved pain, flexibility & disability.

**Conclusion:**

This study revealed that both groups treated by MET and Bowen Technique had significantly reduced pain, improved flexibility of back thigh muscle and reduced functional disability.

## INTRODUCTION

Patients suffering from back pain are unable to perform routine activities. There are many causes that lead to pain in the low back area, such as abnormalities in the vertebra, muscular spasms, and emotional, physical, and biomechanical issues.^1^,^2^ CLBP is often increased by sitting and standing for long periods of time. Postural asymmetry and changes in biomechanics are potential factors that increase the pain threshold.^3^

Hamstring flexibility is described as the capability of back thigh muscles (hamstrings) to increase their length within an available range. Sacroiliac joint dysfunction can be caused by altered movement patterns, pregnancy, trauma, or degenerative changes. Psychological elements can also impact how people experience CLBP. Psychological anxiety can intensify pain signals, making healing difficult.^4^ Functional training is a crucial component of physical therapy for patients with CLBP and hamstring tightness. To improve general function and movement patterns, exercises that match the patient’s daily activities are introduced. Physical therapists use functional training to increase patients’ capacity and achieve better functional results.^5^

Bowen Technique is a gentle, non-invasive bodywork practice that promotes healing and restores body balance. It consists of precise, rolling movements by using fingers and thumbs over certain muscles, tendons, and connective tissues that stimulate the body’s natural healing response. Bowen treatment is considered to relieve stress, improve circulation, and rebalance the autonomic nervous system. It can be applied for fifteen to forty-five minutes.^6^

MET is a manual procedure that uses controlled, voluntary isometric contractions of a target muscle group. MET is claimed to be useful for lengthening a shortened muscle, improving range of motion at a joint, and increasing fluid drainage from peripheral regions. This approach, targeting primarily the soft tissue, is also known as active muscular relaxation.^7^

The primary aim was to discern the relative effectiveness of the Bowen technique and MET in alleviating CLBP, enhancing flexibility, and decreasing disability. The results of this study are anticipated to provide valuable insights into best practices for treating CLBP induced by hamstring tightness.

### Rationale of study:

CLBP is the leading cause of pain in adults. Bowen and MET’s proved to be beneficial for the treatment of CLBP associated with hamstring tightness, but still, there is a need to compare both techniques to find out the best possible treatment.

## METHOD

Study design was Randomized Clinical Trial and conducted at Madinah Teaching Hospital Faisalabad. Duration of Study was four months from February to May 2023. Sample Size was calculated from Open Epi tool using data and Sampling Technique carried out using a non-probability, purposive sampling methodology with sealed envelope method.^8^ The Calculated sample size was 31 in each group. Total Sample size was 74, with a 20% dropout factor added.

### Inclusion Criteria:

Symptoms of patients persisting from more than three months, were included, complaining of pain of ≥ 5 on NPRS and age between 25 to 50 years.^9^ Patients with 20° to 50° loss of active knee extension with hip flexion at 90° 10 and participants who were willing to take part in study.

### Exclusion Criteria:

Patients with disc pathology, radicular pain, acute low back pain, history of spinal fracture or surgery, spondylolisthesis, any systemic disease or TB of spine were excluded. Subjects that had received physical therapy treatment for LBP in a period of six months were also excluded.^11^

### Data Collection Procedure:

It was a single blinded Randomized clinical trial. Participants with low back pain were screened and evaluated through history taking and physical examination. Then participants who fulfill the selection criteria were recruited in the study. Informed consent was taken after whom the subjects were randomly divided into two groups through lottery method, where both groups were experimental and depending upon the allocated sequence and scheduled time period in treatment plan provided. Group-A was treated with Bowen technique and Group-B was treated with MET. Measurements were taken at baseline, at the end of 2^nd^ week and 4^th^ week. There were total of three sessions per week for four weeks i.e. 12 sessions.

### Group-A (Bowen Technique):

- First of all, hot pack was applied at the Hamstring muscle and Tens (high frequency 100 Hz pulse duration 60s 10 minutes) was applied at lower back along both were considered as a routine physical therapy.
- The initial step of the Bowen treatment involved placing the thumb on the surface of the specific muscle.
- The thumb was applied with gentle pressure along the muscle’s lateral edge.
- As the thumb was gradually pressed inwards towards the center, the muscles exhibited various responses, such as being pulled, plopped, or demonstrating other observable reactions.
- Manipulate the skin and engage the muscle through controlled movements.
- The sequence of treatment began with the thumbs and progressed to the fingers.
- Each session of treatment lasted for a duration of 30 minutes

### Group-B (Muscle Energy Technique):

- First of all, hot pack was applied at the Hamstring muscle and Tens (high frequency 100 Hz pulse duration 60s 10 minutes) was applied at lower back along with the routine physical therapy.
- In this technique, the initial step involved extending the subject’s knee until they reported experiencing discomfort in the hamstring.
- Following that, a five-second period of moderate isometric contraction of the hamstring muscle was applied.
- A three-second grace time was allowed.
- Each session of treatment lasted 30 minutes.

### Consent taking:

Participants were asked for their written, informed consent. The data’s confidentiality was guaranteed. The intervention in this study had no drawbacks to the participants. If a participant desired ongoing care, he or she could leave the study at any time with no consequences.

### Outcome measures:

Numeric pain rating scale (NPRS) was used to record the subject’s back pain scores.^12^ Subjects were asked to mark a point on this line according to the intensity of their pain, indicating their pain level. The NPRS exhibited moderate reliability (ICC = 0.67; [0.27 to 0.84]). Active knee extension test (AKET)^13^ measures the active range of knee extension in the hip flexion position. It was measured with goniometer. The intra-class correlation coefficients (ICC2, 1) for inter-rater reliability were .887~.986 for the right knees and .915~.988 for the left knees. Furthermore, the inter-rater (test-retest) reliability (ICC3, 1) values for Raters 1 and 2 were .820~.915 and .820~.884, respectively.

Disability Index having 10 item questionnaire^14^ regarding ADLS performance was filled by patients with each question ranging in score from 0-5. The ODI covers a broader idea of impairment than simply pain severity. Test-retest reliability has been demonstrated to be strong; one research observed values ranging from = 0.83 to 0.99.^15^ All tools have good reliability and validity. The hot pack and TENS (high frequency 100 Hz) was applied on hamstring muscle.

### Ethical approval:

It was obtained from the university ethical committee vide letter number TUF/Addl Reg /SB/358 from the Board of Advance Studies & Research The University of Faisalabad on 28 Jan 2023. This trial is registered in Iranian Registry of Clinical Trial vide number IRCT20230426057994N1.

## RESULTS

Sixty-two individuals were included in this study that was randomly divided into two equal groups, each of 31 participants, by means of the purposive sampling technique. Group-A received Bowen technique, and Group-B received MET. The mean and standard deviation were calculated for quantitative variables (age, gender, etc.). The mean age of the patients in Group-A was 36.612 ± 6.448. The mean age of the patients in Group-B was 37.064 ± 6.697.

The normality of the data was assessed by using the Kolmogorov-Simonov test. The data violates the assumption of normality, so for the analysis of NPRS, AKE and ODI non-parametric tests were applied. The Friedman test was used to determine the intragroup comparison between baseline and post-intervention data for the outcome variable (NPRS, Active Knee Extension Test, Oswestry Low Back Disability Questionnaire). The Mann-Whitney U test was used to determine the between-group comparison.

A P-value-value less than 0.05 was considered significant. Table-I shows the tests statistics of Friedman test for within Group-Analysis of NPRS, AKE and ODI. A statistical significant difference was found in pre and post treatment value of all variable in Group-A as well as in Group-B. Table-II displays the test statistics of Mann Whitney U test for Group-A and B at baseline. The p-value is greater than 0.05, there is no statistical difference in both groups on NPRS, AKE and ODI at baseline. Table-III displayed the test statistics of Mann Whitney U test for Group-A and B after the treatment of four weeks. The p-value is greater than 0.05, there is no statistical difference in Group-A and B on NPRS, AKE and ODI at 4^th^ week.

**Table-I:** Within Group-Analysis of NPRS, AKE, ODI (Friedman Test).

		N	Mean	Std. Deviation	Median	Asymp. sig
Group-A (NPRS)	At Baseline	31	6.35	1.08	6.00	<0.001
	At 2nd Week	31	3.58	1.11	3.00	
	At 4th Week	31	2.03	0.48	2.00	
Group-B (NPRS)	At Baseline	31	6.38	1.11	6.00	<0.001
	At 2nd Week	31	3.87	1.05	4.00	
	At 4th Week	31	2.09	0.39	2.00	
Group-A (AKE)	At Baseline	31	67.25	7.39	70.00	<0.001
	At 2nd Week	31	59.19	6.72	60.00	
	At 4th Week	31	47.25	7.28	50.00	
Group-B (AKE)	At Baseline	31	67.09	7.93	70.00	<0.001
	At 2nd Week	31	57.90	7.72	60.00	
	At 4th Week	31	47.58	7.28	50.00	
Group-A (ODI)	At Baseline	31	45.29	7.95	45.00	<0.001
	At 2nd Week	31	30.90	8.00	31.00	
	At 4th Week	31	20.74	5.25	19.00	
Group-B (ODI)	At Baseline	31	47.41	7.83	48.00	<0.001
	At 2nd Week	31	29.93	8.73	29.00	
	At 4th Week	31	20.00	4.87	19.00	

NPRS: Numeric pain rating scale, AKE: Active Knee Extension, ODI: Oswestry Disability Index.

**Table-II:** Between Group-Analysis of A and B at baseline (Mann Whitney U test).

	NPRS at Baseline	AKE Test at Baseline	ODI at Baseline
Mann-Whitney U	473.000	477.500	406.500
Wilcoxon W	969.000	973.500	902.500
Z	-.109	-.043	-1.043
Asymp. Sig. (2-tailed)	.913	.966	.297

NPRS: Numeric pain rating scale, AKE: Active Knee Extension, ODI: Oswestry Disability Index.

**Table-III:** Between Group-Analysis of A and B at 4^th^ week (Mann Whitney U test).

	NPRS at 4^th^ week	AKE Test at 4^th^ week	ODI at 4^th^ week
Mann-Whitney U	453.500	460.500	460.000
Wilcoxon W	949.500	956.500	956.000
Z	-.553	-.289	-.289
Asymp. Sig. (2-tailed)	.581	.772	.772

NPRS: Numeric pain rating scale, AKE: Active Knee Extension, ODI: Oswestry Disability Index.

## DISCUSSION

Results of the present study revealed that applying Bowen technique and MET on hamstring muscle found to be equally effective in reducing the back pain, improving hamstring muscle flexibility and reducing the disability after the treatment of four weeks. Kage et al. determined the impacts of the Bowen technique compared with MET on 48 participants. The findings of that study supported the results of present study in such a way that both techniques were found to be effective in improving the flexibility and range of mobility of hamstring muscle. But in present study no significant difference was observed in the effects of Bowen technique and MET, in contrast to this Kage et al. reported that the group treated with the Bowen approach showed greater improvement in hamstring flexibility and range of movements. At the end of the third treatment session, the muscular energy technique group performed better in terms of improving hamstring muscle strength.^8^

In present study, findings revealed that Muscles energy technique showed beneficial effects in alleviating the back pain, improving the hamstring range of flexibility and reducing disability in the patients of back pain after the treatment of four weeks. But in contrast to this a systematic review by Franke et al., reported that the quality of studies conducted to assess the effectiveness of MET is limited. Trials are typically very small and have a significant risk of bias due to methodological flaws. To date, studies have provided low-quality evidence that MET is ineffective for people with LBP. There is insufficient evidence to evaluate if MET is likely to be useful in practice.^16^

In present study both Bowen technique and MET on hamstring muscle found to be effective in reducing the back pain, improving hamstring muscle flexibility and reducing the disability after the treatment of four weeks. The beneficial effects of Bowen technique was supported by the study of Marr M et al., who studied the impact of the Bowen Technique on the flexibility of the hamstring. The research found that flexibility levels increased consistently throughout a week.^17^

Patel et al. compared the results of MET with tissue mobilization in subjects who had pain in their backs for more than six months. The research indicated that subjects managed by both of these techniques had equivalent improvements in muscle flexibility (P<0.0001).^14^ The results of that study supported the findings of present study in terms of beneficial effects of METs on improving the hamstring muscle tightness in the patients of low back pain. Another study by Elshinnawy et al. examined the influence of MET compared with taping on ninety subjects who had had back pain for more than six months. It showed that muscle energy and routine physical therapy both reduced pains equally.^18^

This study revealed that MET is effective in increasing ROM and flexibility in CLBP patients. This research supports research by Sojitra N et al. in 2020 to assess the immediate effects of suboccipital muscle inhibition (SMI) and MET in healthy college volunteers. This study concluded that SMI and MET both improve hamstring flexibility.^19^ The recent study was compared with a trial that was conducted by Tanvi A et al. to determine the effects of MET and Mulligan’s bent leg raise (BLR) in patients with hamstring tightness. The outcomes showed that both Mulligans BLR and MET significantly improved the hamstring flexibility in asymptomatic pupils.^20^

### Significance of Study:

With an increasing prevalence of hamstring shortening in patients with CLBP, there is a need to develop a treatment protocol that is valid and effective. This study will help the therapist evaluate the effect of MET and the Bowen Technique on enhancing hamstring muscle flexibility in patients with CLBP due to hamstring tightness. This research will provide more awareness about Bowen therapy and also serve as a benchmark for clinicians conducting similar studies.

The key strength of this research is its randomized design and the use of outcome measures, which enhance the reliability and comparability of the results. The study also demarcates a standard treatment protocol across the groups, allowing for a more direct comparison of the Bowen technique and MET.

### Limitation:

The sample size is relatively small, potentially affecting the generalizability of the results. Additionally, the study focuses on a specific demographic, which may limit the applicability of the findings to a broader population with varied etiologies. Moreover, there was always a chance of patients misreporting the symptoms.

## CONCLUSION 

Both techniques (Bowen technique and MET) were found to be equally effective in terms of decreasing pain and improving muscle flexibility and in reducing disability. Subjects can perform the occupational activities freely by receiving treatment with these methods. In future, increase sample size of the patients and more demographic features can be used. Therefore, better outcomes can be achieved and the research can be used at a wider range.

### Authors’ Contributions:

**KB:** Concept and study design, literature search and literature review, acquisition of data, drafting the manuscript. She is responsible for the accuracy or integrity of the work.

**MM:** Overall supervision, drafting the manuscript, analysis and interpretation of data. Final approval of the version to be published.

**MJ:** Literature search and literature review, critical revision.

**MG:** Concept and study design, critical revision.
